# Revitalizing actinobacteria research: an urgent response to the antimicrobial resistance crisis

**DOI:** 10.1007/s13659-025-00587-8

**Published:** 2026-02-05

**Authors:** Samuel Paulo Cibulski, Valnês da Silva Rodrigues-Junior

**Affiliations:** 1 FACISA–Faculdade de Ciências da Saúde do Trairi, Universidade Federal do Rio Grande do Norte (UFRN), Santa Cruz, Rio Grande do Norte Brazil; 2 Laboratory of Biotechnology in Microorganisms, Biotechnology Center, Universidade Federal da Paraíba (UFPB), João Pessoa, Paraíba Brazil

**Keywords:** Antimicrobial resistance (AMR), Actinobacteria, Antibiotic discovery, Natural products, Bibliometric analysis

## Abstract

**Abstract:**

The crisis of antimicrobial resistance (AMR) is escalating while the antibiotic pipeline remains stagnant. Our bibliometric analysis of eight decades of literature reveals a critical imbalance: research on AMR has grown, yet fundamental research on antibiotic discovery has declined. Most strikingly, research attention to *Actinomycetota*, the source of most clinical antibiotics, has sharply decreased since its mid-twentieth-century peak. This therapeutic disinvestment coincides with the intensifying AMR crisis. We argue for a strategic reinvestment in natural product discovery, now enabled by advances in genomics, artificial intelligence, and synthetic biology. These tools can unlock the vast, silent biosynthetic potential of actinobacteria, transforming discovery into a targeted and efficient endeavor. Rebalancing research priorities by coupling this historically proven source with modern technology is essential to revive the antibiotic pipeline. We urge funding agencies and industry to bridge the growing gap between a well characterized problem and a neglected solution.

**Graphic abstract:**

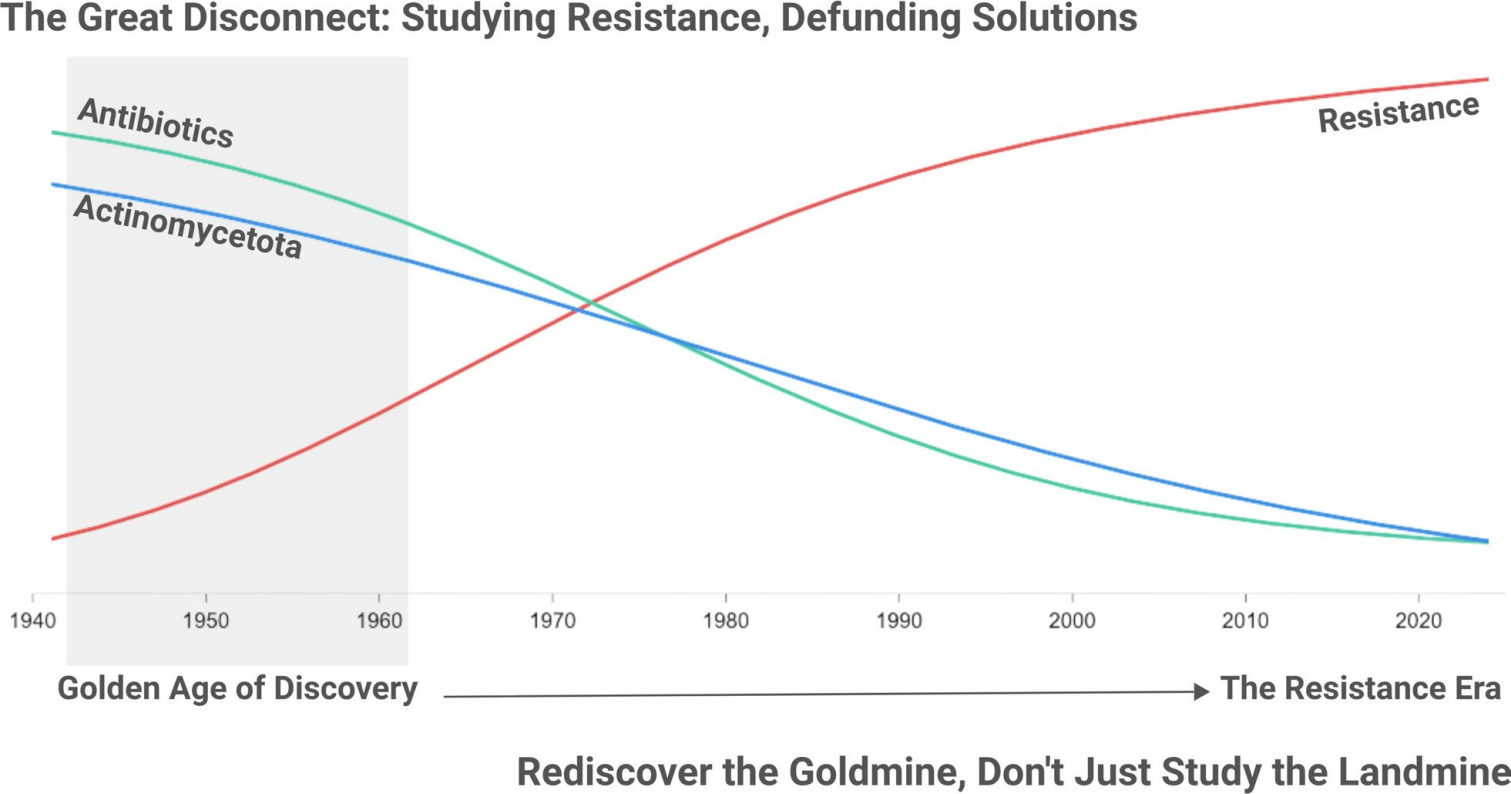

## Correspondence

**Fig. 1:**
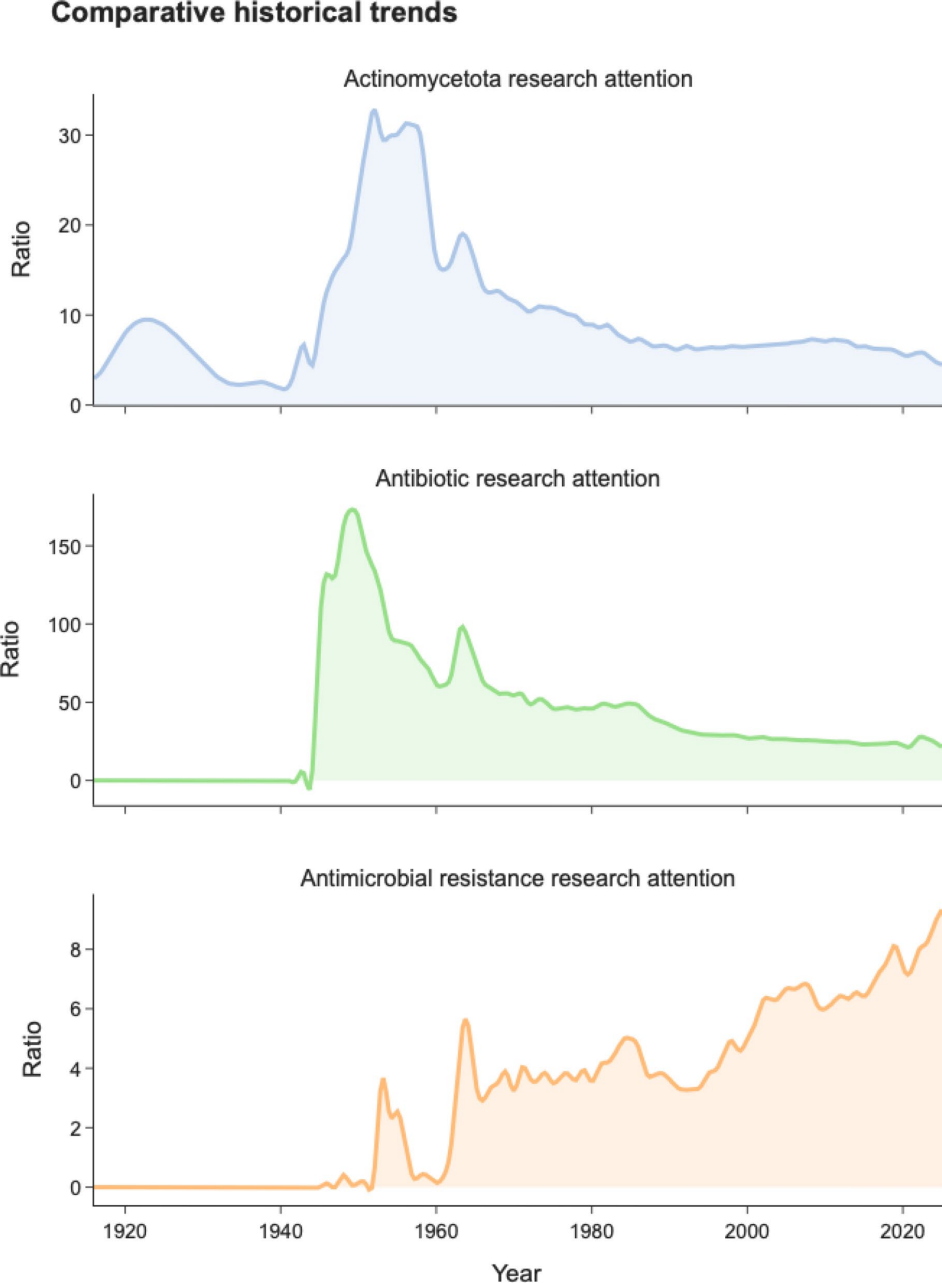
Diverging research priorities in bacteriological literature. (Top) Relative research attention to *Actinomycetota*. Publication data were obtained from PubMed using the search query: “actinobacteria OR actinomycete OR actinomycetota”. Annual counts were normalized against the total number of publications retrieved for the control term “bacteria” each year to calculate the research share (%). The data reveal a 75–80% decline from mid-twentieth century levels. (Middle) Trends in antibiotic and antibacterial research. Data from PubMed using the search query: “antibiotic OR antibacterial”, normalized using the same methodology described earlier. (Bottom) Growth in antimicrobial resistance research. Data from PubMed using the search query: “antimicrobial resistance OR bacterial resistance”, normalized using the same methodology described earlier

The devastating global burden of AMR, with recent studies confirming millions of annual deaths, underscores an accelerating health emergency [1]. Concurrently, WHO warnings about the stagnant antibiotic pipeline highlight our collective failure to address this escalating threat [2]. To investigate the roots of this failure, we conducted a bibliometric analysis of eight decades of PubMed data, which reveals a profound and concerning disconnect in the scientific community’s response to this crisis (Fig. 1).

Our findings demonstrate that that research focus on *Actinomycetota*, the source of approximately two-thirds of all clinically used antibiotics [3], has plummeted from peak values of 15–32% in the 1950s–60s to just 5–6% in recent years, representing a striking 75–80% relative decrease. This is not an isolated trend but part of a systemic shift, as broader antibiotic research has declined in parallel.

Most alarmingly, this therapeutic disinvestment has occurred alongside growing focus on the resistance problem itself. While publications on “antimicrobial resistance” have rightfully increased, reaching 7–8% of bacteriological research, investment in the solutions has declined. We are meticulously documenting the problem while defunding one of its most promising solutions.

This divergence is especially troubling given that the path forward requires a strategic reinvestment in natural product discovery, powered by the very innovations that promise to overcome historical bottlenecks. The exploration of underexplored ecological niches, coupled with genome mining, reveals that the vast majority of actinobacterial biosynthetic potential remains untapped [4–6]. Novel bioinformatic approaches and rapid structural elucidation techniques can now dramatically shorten the discovery timeline, transforming the pursuit of novel antibiotics from a dwindling field into a targeted, high-throughput endeavor.

This vast, untapped potential is now more accessible than ever, thanks to a convergence of breakthrough technologies. Advanced genomics and metagenomics allow us to sequence entire microbial communities from extreme environments, directly accessing biosynthetic gene clusters without cultivation [7–9]. Artificial intelligence and machine learning models can predict the structure and function of novel antimicrobial peptides encoded by these clusters, prioritizing the most promising candidates for synthesis [10, 11]. Furthermore, synthetic biology enables the refactoring and heterologous expression of these complex pathways in tractable hosts, turning silent genetic potential into tangible compounds [12].

Beyond discovery, innovative delivery systems, such as nanoparticle encapsulation or phage-based delivery [13, 14], coupled with rapid diagnostic tools [15] are emerging to enhance the efficacy and responsible use of new antimicrobials. Promising research avenues include targeting bacterial virulence or resistance mechanisms themselves, and the sustainable development of antimicrobials through bioprospecting guided by ecological and evolutionary principles [16, 17].

A practical strategy to accelerate deployment involves fostering open-access genomic databases, public–private partnerships for high-risk discovery, and integrating AI-driven platforms with high-throughput robotic screening. The goal is to create a virtuous cycle: using modern tools to efficiently mine the richest historical source of antibiotics, thereby revitalizing the pipeline with structurally novel, resistance-breaking compounds.

In light of this divergence between a growing problem and a neglected solution, the stagnation is not merely a scientific challenge; it is the result of a quantifiable shift in research priorities. Rebalancing this scale by coupling historical wisdom with modern technology is the imperative first step towards reviving the antibiotic pipeline.

We urge funding agencies and pharmaceutical developers to recognize this imperative. Strategic initiatives that leverage these modern tools to revitalize the discovery of natural products are essential to bridge the dangerous gap between problem and solution.

## Data Availability

All data generated or analyzed during this study are included in this published article.
